# Tumour macrophages as potential targets of bisphosphonates

**DOI:** 10.1186/1479-5876-9-177

**Published:** 2011-10-17

**Authors:** Thea L Rogers, Ingunn Holen

**Affiliations:** 1 Academic Unit of Clinical Oncology, School of Medicine and Biomedical Sciences, University of Sheffield, Beech Hill Road, Sheffield, South Yorkshire, S10 2RX, UK

**Keywords:** Bisphosphonates, macrophages, zoledronic acid, tumour microenvironment, tumour-associated macrophages, anti-tumour effect, mevalonate pathway

## Abstract

Tumour cells communicate with the cells of their microenvironment via a series of molecular and cellular interactions to aid their progression to a malignant state and ultimately their metastatic spread. Of the cells in the microenvironment with a key role in cancer development, tumour associated macrophages (TAMs) are among the most notable. Tumour cells release a range of chemokines, cytokines and growth factors to attract macrophages, and these in turn release numerous factors (e.g. VEGF, MMP-9 and EGF) that are implicated in invasion-promoting processes such as tumour cell growth, flicking of the angiogenic switch and immunosuppression. TAM density has been shown to correlate with poor prognosis in breast cancer, suggesting that these cells may represent a potential therapeutic target. However, there are currently no agents that specifically target TAM's available for clinical use.

Bisphosphonates (BPs), such as zoledronic acid, are anti-resorptive agents approved for treatment of skeletal complication associated with metastatic breast cancer and prostate cancer. These agents act on osteoclasts, key cells in the bone microenvironment, to inhibit bone resorption. Over the past 30 years this has led to a great reduction in skeletal-related events (SRE's) in patients with advanced cancer and improved the morbidity associated with cancer-induced bone disease. However, there is now a growing body of evidence, both from *in vitro *and *in vivo *models, showing that zoledronic acid can also target tumour cells to increase apoptotic cell death and decrease proliferation, migration and invasion, and that this effect is significantly enhanced in combination with chemotherapy agents. Whether macrophages in the peripheral tumour microenvironment are exposed to sufficient levels of bisphosphonate to be affected is currently unknown. Macrophages belong to the same cell lineage as osteoclasts, the major target of BPs, and are highly phagocytic cells shown to be sensitive to bisphosphonates in model studies; *In vitro*, zoledronic acid causes increased apoptotic cell death; *in vivo *the drug has been shown to inhibit the production of pro-angiogenic factor MMP-9, as well as most recent evidence showing it can trigger the reversal of the TAMs phenotype from pro-tumoral M2 to tumoricidal M1. There is thus accumulating evidence supporting the hypothesis that effects on TAMs may contribute to the anti-tumour effect of bisphosphonates. This review will focus in detail on the role of tumour associated macrophages in breast cancer progression, the actions of bisphosphonates on macrophages *in vitro *and in tumour models *in vivo *and summarise the evidence supporting the potential for the targeting of tumour macrophages with bisphosphonates.

## Introduction

Breast cancer is the most commonly diagnosed cancer in the UK where women have a 1 in 8 lifetime risk of developing the disease [1]. The majority of breast cancer patients will present with a localised tumour, however at least 5% of patients will present with advanced metastatic disease, and it is estimated that a further 30% will go on to develop this within 10 years. The most common site of metastatic spread is bone, occurring in approximately 80% of advance disease patients. The consequences of bone metastases include bone pain, pathological fractures and hypercalcaemia, - collectively known as skeletal-related-events (SREs) have decreased over the past 30 years; this is mainly to the introduction of bisphosphonates as part of standard advanced breast cancer treatment. This widespread use has lead to increasing interest in the potential for the bisphosphonates to affect tumour growth, both as a consequence of reduced bone resorption but also through actions on tumour cells and cells of the tumour microenvironment, including macrophages [2].

### Macrophages in the tumour microenvironment

Cancer cells work in conjunction with cells in the surrounding microenvironment to aid numerous processes needed for tumour development. Macrophages are a major component of this microenvironment, and are of particular interest as potential therapeutic targets due to their central role in tumour progression.

**Figure 1:**
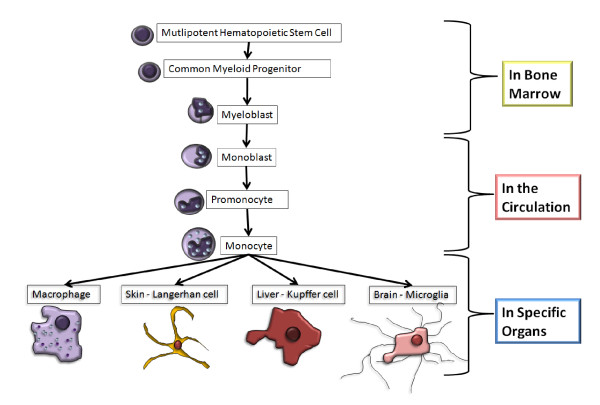
**Development of different types of macrophages from multipotent hematopoetic stem cells**.

Macrophages are lymphocytes of the myeloid lineage, derived from CD34+ bone marrow progenitor cells (see Figure 1) [3,4]. Pro-monocytes develop into monocytes in the bloodstream and can then either circulate as inflammatory monocytes, that differentiate into macrophages in inflamed tissue, or extravasate into tissues and differentiate into resident macrophages [3,4]. Resident macrophages have different phenotypes depending on the tissue they reside in, for example: Kupffer cells in the liver, microglia in the brain and Langerhan cells in the skin. Both types of macrophages, inflammatory and resident, are phagocytes, and both perform a range of essential biological functions [3-6].

Macrophages possess phenotypic plasticity that can be classified into two types, M1 (Type I) and M2 (Type II) polarised macrophages. These have different characteristics and functions within the body and immune system; shown by the varying types and amounts of cytokines they produce (see Table 1) [3-6].

**Table 1:** Difference between M1, M2 and TAM activation, membrane receptors, cytokines/chemokines produced and markers.

	M1-Classically Activated	M2-Alternatively Activated	TAMS	References
**Activation**	INFγ, LPS	IL-4, IL-13, IL-10	CSF-1, VEGF, CCL2, CCL3, CCL4, CCL5, CCL8, MCP-1, IL-4, IL-13, IL-10, TGFβ-1, PGE_2_.	Coffelt *et al *[4] Joyce and Pollard [9] Mantovani *et al *[84]

**Membrane Receptors**	TLR2, TLR4, CD16,CD32, CD64, CD80, CD86	Scavenger receptor A, Scavenger receptor B, CD14, CD23, CD163	CD11b^+^, CD14^-^, CD31^-^, CD45^+^, CD68^+^, CD117^-^, CD122^-^, CD146^-^, CD204^+^, CD206^+^, CCR2^+^,CSF1R^+^, MHCII^+^, CD23^+^, CD163^+^, CXCR4^+^, VEGFR1^+^, VEGFR2^-^, F4/80^+^(mice)	[4,9,84]

**Cytokines produced**	IL-1, IL-6, IL-12, TNF, RNI, ROI	IL-1ra, IL-1 decoy receptor, EGF, FGF, VEGF, TNF-β,	bFGF, FGF, HFG, EGFR, PDGF, VEGF, ANG1, ANG2, IL-1, IL-8, TNF-α, TP, MMP-2, MMP-2, MMP-9, NO, CSF-1	[4,9,84]

**Chemokines produced**	CCL-2, CCL-3, CCL-4, CCL-5 CXCL8, CXCL9, CXCL10, CXCL11	CCL-12, CCL-16, CCL-17, CCL-18, CCL-22, CCL-24	CCL-2, CCL-3	[4,9,84]

**Marker**	iNOS	Arginase	F4/80 (mice), CD34 (humans)	[4,9,84]

M1 macrophages, also known as classically activated macrophages, play various roles in both arms of the immune system. In the innate immune system they guard against infection by engulfing and digesting invading microbes, as well as defending against tumour cells by releasing cytotoxic nitric oxide and reactive oxygen intermediates. In the adaptive immune system they operate as lymphocyte activators by presenting antigens to polarised type I T cells and secreting immunomodulatory and proinflammatory cytokines [3-6].

M2 macrophages (also known as alternatively activated macrophages) are better adapted to scavenging debris, and secrete growth factors that promote angiogenesis. They show reduced immune activity such as poor antigen-presenting capabilities and suppress T cell and natural killer cell proliferation and activity. They are still highly phagocytic but mainly help repair sites of injury by engulfing cell debris, regulating tissue remodelling and repair and control normal cell turnover [3,6]. M2 macrophage classification can be broken down further into M2a, M2b and M2c classes depending on environmental signals that induce their activation, with M2c being the more immunosuppressive of these phenotypes [7,8]. However it must be noted that there is a degree of overlap between the different types of macrophages and separation of them is neither easy nor clear-cut.

### Tumour associated Macrophages (TAMs)

The tumour stroma plays a central role in tumour progression. Via cellular and molecular interactions between the stroma and the tumour cells, the stoma is able to change with the tumour as it evolves [9]. Cells of the tumour stroma include macrophages, fibroblasts, lymphocytes, other bone marrow derived cells, pericytes, myeloid-cell derived suppressor cells, mesenchymal stem cells and blood and lymphatic vessels [5].

Primary and secondary tumours are associated with chronic inflammation leading to recruitment of bone marrow derived cells, macrophages are a major component of this inflammatory infiltrate and thus of the tumour stroma itself [5,10]. Tumours are capable of altering the function of many biological systems, the prime example being the "hijacking" of this inflammatory infiltrate to aid tumour progression. Pre-invasive tumour cells release chemotactic factors that attract circulating monocytes into the tumour stroma. In breast cancer these factors include, colony stimulating factors (CSF-1) [11], vascular endothelial growth factor (VEGF) [12] and many CC chemokines such as CCL2, CCL3, CCL4, CCL5 and CCL8, monocyte chemotactic protein - 1 (MCP-1) [6,13,14], see Table 1 for details.

Once in the tumour stroma the macrophages differentiate into tumour-associated macrophages (TAMs). It has been proposed that exposure to the tumour cells and to additional tumour-derived molecules such as IL-4, IL-10, IL-13, TGFβ-1 (transforming growth factor - β) and PGE2 (Prostaglandin E_2_) initiates the development of TAMs, as well as initiating the development of characteristics akin to those of the M2 macrophages [3,6]. The "M2 polarised macrophage characteristics" TAMs develop are advantageous to the tumour as they support growth, invasion, migration and metastatic spread. TAMs also produce a wide range of pro-angiogenic and immunosuppressive factors. In other words, macrophages are recruited into the tumour microenvironment and then "educated" to regulate inflammation and support the progression of the tumour [5]. These activities will be discussed in more detail below.

**Figure 2:**
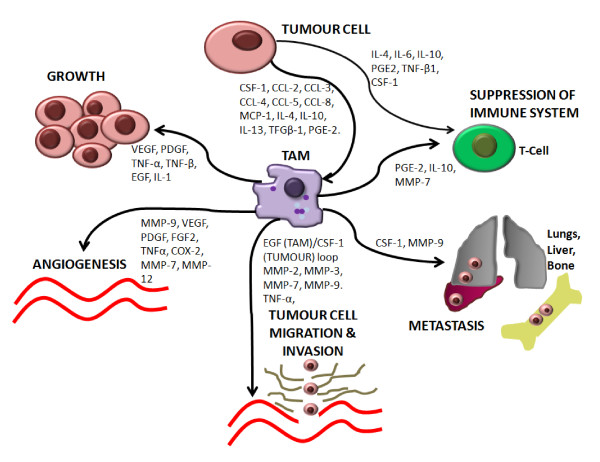
**Role of tumour associated macrophages in tumour progression**.

It is generally accepted that TAMs have mostly pro-tumoral functions [5] and play an important role in several stages of tumour progression. This progression involves a series of events that leads from the primary site to the metastatic site, including tumour cell growth, angiogenesis, migration, invasion, intravasation and finally extravasation at distant site where the process begins again (metastasis). Simultaneous immunosuppression is also needed to facilitate this process, as this allows cancer cells to evade detection by immune cells and therefore travel unharmed in the circulation where they adhere and extravasate at distant sites. Macrophages act like the "Jack-of-all-trades", being involved in all of these processes described in the following sections (Figure 2) [15,16].

#### The role of TAMs in angiogenesis

Angiogenesis is a key step in tumour progression, without which, tumours would not be able to survive and progress. Only tumours with a maximum of 2 mm diameter can be perfused by simple diffusion and anything larger than this needs an additional blood supply [17]. The change in tumour phenotype from angiostatic to angiogenic is known as the angiogenic switch, and is a prerequisite for the progression of the tumour to a malignant state and for its metastatic spread [10,17]. Angiogenesis is an intricate process involving synchronised basement membrane degradation and endothelial cell proliferation and migration [18]. There is a wealth of research linking tumour-associated macrophages to increased angiogenesis, with many published reports focussing on the molecular mechanisms of this process [6,12,17,19-21].

TAMs gather in avascular tumour "hotspots", areas of necrosis with few blood vessels, and there is an inverse relationship between macrophage density and vascular density [19]. Angiogenesis is triggered by tumour hypoxia; where an area of tissue is deprived of an adequate oxygen supply. The low oxygen tension up-regulates the expression of certain chemoattractants including VEGF and endothelins EL-1 and EL-2, which attracts TAMs into hypoxic tumour sites where they are then immobilised by down-regulating chemoattractant receptors [6,22]. Hypoxia also causes increased expression of factors encoding for pro-angiogenic genes, e.g. hypoxia-inducible transcription factors HIF-1 and HIF-2 [23]. These up-regulate TAM production of pro-angiogenic growth factors and cytokines such as matrix metalloproteinase-9 (MMP-9), vascular-endothelial growth factor (VEGF), platelet-derived growth factor (PDGF), fibroblast growth factor 2 (FGF2), tumour necrosis factor-α (TNF-α), COX-2, MMP-7, MMP-12 [6]. TAMs and tumour cells work together in a series of paracrine loops. For example, TAMs produce of IL-1 which induces HIF-1 expression by the tumour and this is known to up-regulate the production of VEGF by TAMs [17]. TAMs also work in autocrine loops, and TAMs in poorly vascularised breast cancers are shown not only to express VEGF but also to respond to it [12]. VEGF is a pro-angiogenic factor that stimulates the proliferation and migration of endothelial cells needed for capillary formation. Inhibiting VEGF-A expression with bevacizumab, (a fully human anti-VEGF antibody) in SCID mice with established orthotopic MDA-MB-231 breast tumours caused reduced TAM infiltration which was correlated with reduced microvessel density and reduced VEGF-induced angiogenesis [20]. TAMs also produce matrix-metalloproteinases such as MMP-7 and MMP-9. MMP-9 is a stromal factor essential for angiogenesis; it remodels the extracellular matrix and promotes the sprouting and growth of new blood vessels by making VEGF available to appropriate receptors on endothelial cells [21]. MMP-7 promotes endothelial cell proliferation and migration thus supporting angiogenesis [6].

A study using CSF-1 null mutant PyMT mice showed that macrophage infiltration was a prerequisite for the angiogenic switch which correlates with the transition of the tumour phenotype to malignancy [11]. This is supported by other studies in different cancers including: oral squamous cell carcinoma, where it was found that increased TAM infiltration was associated with higher histopathological grade [24], as well as in malignant melanoma [25], where a positive correlation between mean macrophage count and mean vascular count was found [11,24,25].

#### The role of TAMs in tumour growth

TAMs are evidently multifunctional and can influence tumour growth both indirectly and directly [6]. The former, as described above, is mediated through their role in angiogenesis, which is essential for tumour growth as it provides oxygen and nutrients [18]. However they are also more directly involved; TAMs secrete a number of mitogenic cytokines and growth factors that are involved in a range of paracrine loops which lead to proliferation of tumour cells and thus growth of the tumour [6,18,26]. These cytokines and growth factors include: platelet derived growth factor (PDGF), TNF-α and transforming growth factor - beta (TGF-β), epidermal growth factor (EGF) and IL-1 [6,14]. There are a number of studies showing that TAM infiltration correlates with increased tumour cell proliferation and thus increased growth of many tumours, including breast cancer [27].

*In vitro *studies have shown that growth of malignant lymphoma cells is regulated by macrophage-like stromal cells: when highly malignant murine RAW117-H1 cells were grown on a layer of J774A.1 (Balb/c macrophage cell line), direct cell surface contact between the stromal and lymphoma cells was needed for growth regulation [28]. It has also been indicated that macrophage cell surface components act synergistically with FIO 30 cells (another murine lymphoma cell line) to support tumour cell growth, only when cells were in close proximity [29]. It would be of interest to determine if this cell-to-cell contact is necessary in other cancers, especially breast cancer. Evidence that TAMs support tumour growth has also been reported in murine sarcoma cells (MC1 cell line) which only grew *in vitro *when co-cultures with peritoneal macrophages [30]. Moreover, the inhibition of macrophage infiltration by transfecting IL-10 into Chinese Hamster Ovary cells, suppressed subsequent tumour growth [31].

A review by Leek *et al *described how TAMs -that secrete the majority of EGF in tumour stroma, preferentially stimulated the breast tumour cells that express EGF-receptors thereby creating a predominantly EGFR-expressing tumour, which is correlated with poor survival [14]. This process seems analogous to environmental pressure and shows that, not only do tumours influence the TAM phenotype, but TAMs can influence the overall tumour phenotype. Conversely there is also evidence that TAMs can delay tumour growth by secretion of factors such as nitric oxide and interferon gamma [32], supporting the view that different areas of the tumour can activate different TAM phenotypes [6].

#### The roles of TAMs in migration, invasion and intravasation

Several reports have proposed a chemotactic and paracrine EGF/CSF-1 loop between macrophages and tumour cells, which allows them to work synergistically in an effort to co-migrate. Macrophages express CSF-1 receptors and produce EGF, whereas tumour cells express EGF receptors and produce CSF-1. EGF stimulates the migration of tumour cells and up-regulates the production of CSF-1 which subsequently promotes migration of TAMs. Thus the two cell types migrate together in a co-dependent manner. This supports findings by Lin *et al *who showed reduced infiltration of macrophages into tumours in CSF-1 null mutant PyMT mice and thus, reduced invasion by tumour cells [11].

Moreover, EGF and CSF-1 induce the formation of invadopodia (in metastatic mammary adenocarcinoma cells) and podosomes (in TAMs), respectively. Both are involved in extra-cellular matrix (ECM) degradation and remodelling, thus increasing invasion of both cell types [33-37]. In *in vivo *models of breast cancer, this migratory response is concurrent with invasion, intravasation and metastasis [37].

Invasion of a tumour through the basement membrane is the point at which it is classified as malignant and marks the start of the metastatic cascade. TAMs, as well as forming podosomes, are capable of secreting factors that break down areas of basement membrane, thus allowing movement of tumour cells into the surrounding tissues. These include matrix metalloproteinases in particular MMP-2, -3, -7 and -9 [37,38], enzymes involved in ECM remodelling and thus, aid invasion. MMP production by macrophages is stimulated by TNF-α, a cytokine produced by tumour cells. *In vitro *studies have shown that co-culture of breast cancer cells and macrophages up-regulated MMP expression in macrophages in a TNF-α dependent fashion, causing enhanced invasiveness of the tumour cells [38]. These findings were verified using a broad spectrum MMP antagonist, which significantly reduced the invasion. Similarly, addition of a TNF-α antibody reduced invasiveness and expression of MMP-2 and MMP-9 by TAMs [38]. However, conflicting findings have been reported on the necessity of cell surface contact discussed above [38].

Migration of tumour cells and TAMs occur in areas of collagen fibrillogenesis and angiogenesis, where both cell types move along collagen fibres attached to blood vessels in a lockstep fashion. Once they reach the blood vessels, macrophage aid tumour cells intravasation into the circulation. In addition, invasion occurs at sites of angiogenesis, and there is also evidence that macrophages promote collagen fibrillogenesis [39].

#### The role of TAMs in metastasis

In contrast to the role of macrophages in primary tumours, little is known about the specific role of TAMs in metastatic foci; the data suggests that macrophages are also involved at the other end of the metastatic cascade, aiding extravasation of tumour cells and the establishment of a proliferative niche. One study showed that when carcinoma cells were injected into the portal vein, mice with depleted peritoneal macrophages had reduced tumour foci in the lungs, indicating that the macrophages were directly involved in seeding of the tumour cells [40].

Studies of the effects of CSF-1 on tumour progression and establishment of metastasis showed that mice deficient in macrophage CSF-1 had delayed metastatic spread of mammary tumours to the lungs. In contrast, CSF-1 deficiency did not affect development or growth of the primary tumour [11]. Tumour cells at primary sites induce the expression of MMP-9 in macrophages in the lungs which promotes angiogenesis, aiding tumour cell establishment and growth at this metastatic site [41]. Tumours in macrophage-depleted animals grew to a large size but remained benign. This supports the theory that macrophages are needed in the angiogenic switch which is linked to metastatic potential [10].

#### The role of TAMs in immunosuppression

As discussed above M1 macrophages play important roles in both arms of the immune system including phagocytosis, antigen presentation and release of pro-inflammatory cytokines and are therefore naturally tumoricidal. In contrast, M2 macrophages show poor immune-stimulatory and antigen presenting capabilities. As would be expected, TAMs possess M2-like traits; Tumour cells and the tumour microenvironment release a variety of factors that facilitate this, including IL-4, IL-6, IL-10, PGE_2_, TGF-β1 and CSF-1, expressed by tumour cells, as well as IL-10, PGE2 and MMP-7 expressed by TAMs. IL-10, PGE2 and TGF-β have been shown to suppress the proliferation and cytotoxicity of T cells and NK cells by decreasing macrophage expression of IL-12. MMP-7 is shown to increase tumour cell resistance to chemotherapeutic agents [42,43] and to decrease tumour cell sensitivity to apoptosis [42,43], both of which increase tumour survival.

Overall the evidence suggests that the down-regulation of the normal immune-response to tumour cells by TAMs allows tumours to grow unchecked as well as aiding metastatic spread [6,15].

#### Correlation of TAMs to prognosis and survival

Due to TAMs extensive involvement in tumour progression, it is no surprise that TAM infiltration of the tumour microenvironment has been correlated with decreased patient survival, especially in breast cancer. Histological analysis of 75 invasive breast cancers by Lee *et al *showed that TAM infiltration was associated with high tumour grade, tumour necrosis and large tumour size [44]. Focal macrophage infiltration is reported to be associated high vascular grade, increased necrosis and decreased relapse-free and overall survival in invasive breast cancer [19]. Moreover a meta-analysis found that, in over 80% of cases of human malignancies, increased TAM density was associated with poor prognosis [18].

This integral role that TAMs play in various processes aiding tumour progression and metastasis, make them an important and potential therapeutic target. One such therapy could be the commonly used anti-resorptive drugs nitrogen-containing bisphosphonates, which have been shown to affect macrophages both *in vitro *and *in vivo *as described in the following sections.

### Bisphosphonates

**Figure 3:**
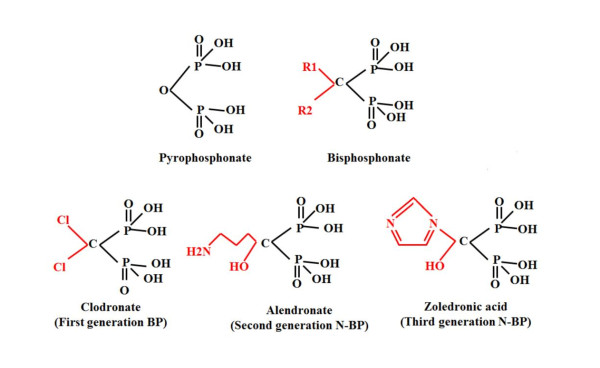
**Structure of pyrophosphonate (top left), general structure of a bisphosphonate (top right), structure of clodronate (bottom left), alendronate (bottom middle) and zoledronic acid (bottom right)**.

Bisphosphonates are stable inorganic analogues of pyrophosphonate in which the central oxygen atom has been replaced by a carbon atom (P-O-P vs P-C-P), see Figure 3. Bisphosphonates were originally used industrially in fertiliser and as anticorrosive agents; however, after the discovery of their ability to inhibit on osteoclast function and hence bone resorption, they were refined for medical purposes. They currently play a fundamental part in the treatment of many metabolic bone diseases including: cancer-induced bone disease, Paget's disease and osteoporosis [45-47].

#### Classification

The P-C-P structure of bisphosphonates forms the central backbone to which two side chains, R1 and R2, are covalently bonded. It is the presence or absence of nitrogen on the R2 side chain that divides bisphosphonates into their two classes; nitrogen-containing bisphosphonates (N-BPs) and non-nitrogen containing bisphosphonates (non N-BPs) [45-47] (see Figure 3).

Non-nitrogen containing bisphosphonates are metabolically converted AppCl2p which inhibits the exchange between ATP and ADP, impairing mitochondrial function and thereby inducing apoptosis [48-50].

**Figure 4:**
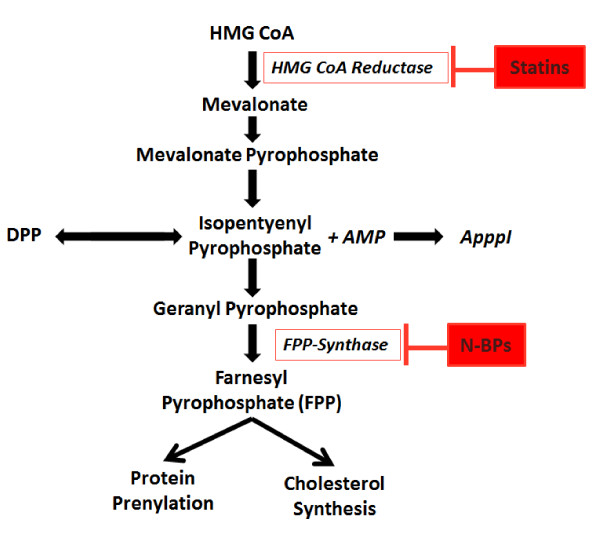
**Schematic diagram of the mevalonate pathway for cholesterol synthesis**. Nitrogen-containing bisphosphonates (N-BPs) inhibit farnesyl diphosphate (FPP) synthase, preventing synthesis of farnesyl diphosphate (FPP) and geranylgeranyl diphosphate (GGPP) required for the prenylation of a number of key proteins essential for cell survival. Inhibition of FPP synthase also causes the accumulation of isopentenyl diphosphate (IPP), which is incorporated into the cytotoxic metabolite ApppI (triphosphoric acid 1-adenosin-5'-yl ester 3-(3-methylbut-3-enyl) ester). Statins also act through this pathway by inhibition of HMG-CoA reductase.

Nitrogen containing bisphosphonates (also known as amino-bisphosphonates or N-BPs) modify protein prenylation by inhibiting farnesyldiphosphonate (FPP) synthase, a key enzyme in the mevalonate pathway, present in all eukaryotic cells [48-52], Figure 4. These mechanisms will be discussed in greater detail below.

#### Mechanism of action of Nitrogen-containing Bisphosphonates

N-BPs can further be classified into second generation N-BPs (pamidronate and alendronate) and the more potent third generation N-BPs (zoledronic acid and risedronate). Third generation N-BPs include a heterocyclic ring at R2, i.e. a nitrogen-containing ring. Both second and third generation N-BPs are much more potent than their non-nitrogen containing predecessors, due to their increased actions on the mevalonate pathway [46,47,52,53].

The mevalonate pathway is responsible for cholesterol synthesis and post-transitional prenylation of a number of molecules including small GTPases such as Ras (and Ras-related proteins e.g. Rap1a). By inhibiting FPP synthase, N-BPs reduce this protein prenylation, thereby inducing apoptotic cell death. They can also, indirectly, increase the synthesis of ApppI (an ATP analogue) which is converted to AMP and IPP, the excess IPP leads to apoptosis, similar to the mechanism of non N-BP's. Production of ApppI is a unique effect of N-BP's. Therefore N-BPs have two methods of inducing apoptosis; inhibition of FPP and excess production of IPP [48-52].

#### Pharmacokinetics

Bisphosphonates are poorly absorbed in the gut due to their negative charge hindering their transport across the lipophilic cell membrane; they are therefore given mainly intravenously [45,54]. BPs are not metabolised and have very short plasma half lives, being distributed quickly to bone or excreted unchanged by the kidneys [45]. Due to their different potencies, there are marked differences in recommended dosing concentrations as well as minor differences in plasma and terminal half-lives. A compilation of clinically relevant pharmacokinetic information is shown in Table 2.

**Table 2:** Bisphosphonates relative anti-resorptive potency, clinical dosage, and route of administration.

Bisphosphonate	Potency	Clinical Dose	Route	**Ref**.
Etidronate	1		Oral, IV	[59]

Clodronate	10	1600 mg/day	Oral	[59,60]

Pamidronate	100	90 mg. 3-4 weeks	IV	[59,94]

Alendronate	1000	10 mg/kg oral. 1 mg/kg I.V)	Oral	[59,62]

Ibandronate	1,000 - 10,000	6 mg 3-4 weeks	IV	[59]

Risedronate	1,000-10,000	30 mg/d	Oral	[59]

Zoledronic acid	100,000	4 mg/2-3 weeks	IV	[3,59,95,96]

As many studies included in this review use zoledronic acid (ZOL), it is important to note the following pharmacological information: The standard 4 mg clinical dose of ZOL administered as an infusion every 3-4 weeks in the treatment of cancer-induced bone disease has a plasma half life of 105 minutes and a peak plasma concentration (C_Max_) of 1-2 μM [55]. Peripheral tissues will therefore receive only low doses of ZOL for short time periods during clinical administration of this agent, whereas many of the *in vitro *and *in vivo *studies that report effects of BPs have used high concentrations and extensive incubation periods.

#### Effects of Bisphosphonates on Osteoclasts

Because bisphosphonates home to bone very quickly due to their high affinity for hydroxyapatite, they are present in the skeleton for prolonged periods [56]. Osteoclasts are the only cell type capable of resorbing bone and will therefore be exposed to BPs bound to the bone matrix, internalising the drug via endocytosis.

Once in the cell cytoplasm the bisphosphonate inhibits protein prenylation, which is vital as the affected signalling proteins are involved in many interactions needed for cell survival, including: membrane ruffling, integrin signalling and endosomal trafficking [56]. Inhibition of prenylation in osteoclasts caused loss of the ruffled border, disruption of cytoskeleton, altered intracellular and extracellular protein signalling as well as induction of apoptosis. Thus bisphosphonates inhibit bone resorption by causing apoptotic osteoclast cell death. This is the basis for their universal clinical use as anti-resorptives f(57).

### Anti-tumour activity of N-BPs - examples from breast cancer

Bisphosphonates are an essential and standard part of breast cancer treatment in the advanced setting, primarily for their activity in bone. In addition, there is a wealth of preclinical evidence showing N-BPs are capable of affecting tumour cells directly, as well as have anti-angiogenic effects [58].

#### Effect on tumour cells in bone

**Figure 5:**
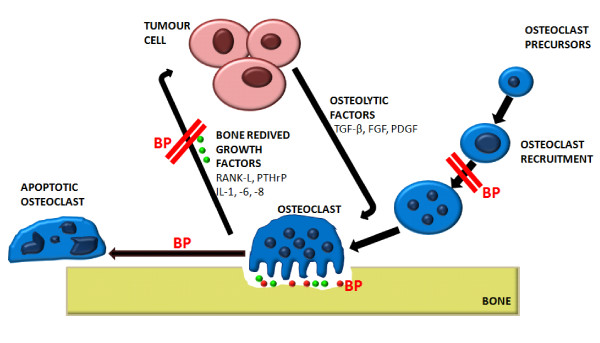
**The Vicious Cycle of Cancer-induced bone disease**.

Breast cancer cells readily metastasise to bone where they release osteolytic factors such as PTHrP, prostaglandin-E and interleukins. These stimulate the production of Receptor Activator of Nuclear Factor κ B ligand (RANKL) which binds to RANK receptors on pre-osteoclasts causing increased osteoclastogenesis and osteoclast activity, thus increasing bone resorption. As bone is resorbed, growth factors such as TGF-β are released which stimulate the proliferation of tumour cells, and the process continues. This process, known as the vicious cycle, is illustrated in Figure 5[59].

It is now well established that bisphosphonates reduce cancer-induced bone disease by interfering with this vicious cycle and osteoclast-mediated bone resorption. This in turn reduces the risk of patients developing a skeletal-related event (SRE) such as bone pain, pathological fractures and hypercalcaemia. On a molecular level, bisphosphonates reduce the release of bone derived growth factors, hence indirectly inhibiting tumour cell proliferation.

#### Direct anti-tumour effects

Evidence from primarily *in vitro *but also some *in vivo *studies have shown N-BPs can decrease tumour cell proliferation, adhesion, migration and invasion, increase apoptosis and decrease angiogenesis. These activities not only lead to a reduction in skeletal tumour burden and hinder the progression of bone metastasis, but may also decrease tumour burden at the primary site.

#### Effects on tumour cell adhesion and invasion

The ability of N-BPs to inhibit tumour cell adhesion in breast cancer was first reported by Van der Pluijm *et al *who pre-treated bovine cortical bone slices with increasing concentrations (1- 100 μM) of etidronate (ETI), clodronate (CLO), pamidronate (PAM), olpadronate, alendronate (ALN) and ibandronate (IBA) prior to seeding of MDA-MB-231 human breast cancer cells. IBA, PAM, ALN and olpadronate (all N-BPs) all dose-dependently inhibited adhesion and spreading of breast cancer cells to bone, whereas ETI or CLO (non N-BPs) had no effect. These findings are supported studies of the effects of IBA, PAM and CLO treatment on subsequent adhesion of MDA-MB-231 and MCF-7 cells to bone, showing that the drugs reduced adhesion with the same order of potency as reported by Van der Pluijm *et al *[60,61]. Bisphosphonate concentrations capable of inhibiting adherence did not induce tumour cell apoptosis, and there was no inhibitory effect on fibroblasts [60].

ZOL, CLO and IBA also showed dose-dependent inhibition of MDA-MB-231 invasion, with the order of potency of the remaining BPs was in concordance with previous results (overall ZOL > IBA > PAM > CLO) [60-62].

*In vivo *studies using murine models have reported inhibition by ZOL on invasion and migration of breast cancer cells. Female BALB/c mice injected with 4T1/luc breast cancer were treated with intravenous ZOL (5 μM/mouse). Histological examination showed a significant decrease in bone, lung and liver metastases in mice that were repeatedly treated (4 times). There was no significant increase in apoptosis of tumour cells in the lungs or the liver, indicating that ZOL had a direct effect at the primary tumour site; therefore it is possible that intensive ZOL treatment can affect migration and invasion of breast cancer cells *in vivo *[63].

#### Effects on apoptosis and proliferation

N-BPs can also induce significant tumour cell apoptosis and inhibit cell proliferation *in vitro*. Investigating the effects of N-BPs on viability and growth of breast cancer cell lines, Fromigue *et al *showed that PAM, IBA and ZOL (0.01-1000 μM) induced cell death mainly via apoptosis in MCF-7 and via necrosis in T47D cells, whereas no apoptosis was seen in MDA-MB-231 cells. PAM, IBA and ZOL also decreased cell growth in a dose- and time-dependent manner, in all cell lines (0.1-1000 μM). ZOL was found to be the fastest acting N-BP, inducing apoptosis within 2 hours (≥ 0.1 μM) in MCF-7 cells and inhibiting cell growth within 3 hours. This rapid effect may be clinically relevant as extra-skeletal exposure to BPs is very brief [64]. These findings were supported by a number of subsequent studies; Senaratne *et a*l found that ZOL and PAM reduced cell growth and viability by 50% in MDA-MB-231 and MCF-7 cells at concentrations of 15 μM and 20 μM (ZOL) and 40 μM and 30 μM (PAM). Moreover these N-BPs induced cell apoptosis in all cell lines [65]. They went on to show ZOL induced apoptosis via inhibition of mevalonate pathway [66], which was supported by Jagdev *et al *who also found that acute exposure to 100 μM of ZOL for just 2 hours caused significant induction of tumour cell apoptosis, which is again, clinically encouraging [64].

### Effects of N-BPs on macrophages

Macrophages and osteoclasts are highly endocytic cells that share the same lineage, so it would be logical that bisphosphonates would affect macrophages. Using macrophage like J774 cells, Thompson *et al *showed that, *in vitro*, bisphosphonates are internalized into vesicles by fluid-phase endocytosis; once inside the cell, endosomal acidification caused the release of the bisphosphonate into the cytosol. They concluded that highly phagocytic cells such as macrophages have the ability to internalise bisphosphonates which makes them ideal targets for these drugs [67]. However, *in vivo*, this would be dependent on the pharmacokinetic properties of the drugs, such as half-life in the circulation, as well as the location of the target cells.

Due to the difficulty of studying osteoclasts *in vitro*, early work on mechanism of action of bisphosphonates was done on macrophage like J774 cells [50]. Bisphosphonates were shown to inhibit macrophage proliferation, migration, invasion, as well as cause apoptosis, supporting that macrophages are potential N-BP targets [49,51,53,56,68-70]. More recent studies have focussed on how bisphosphonates affect "pro-tumoral factors" produced by macrophages and the consequence of this inhibition [21,71,72]. These effects will be covered in more detail below.

#### Effects on apoptosis

While investigating the effects of bisphosphonates on osteoclasts, Rogers *et al *found that pamidronate, alendronate and ibandronate (100 μM/24 h) could inhibit proliferation, reduce cell viability and cause apoptotic cell death of macrophage-like J774 and RAW264 cells [68]. They subsequently discovered that apoptosis is due to nitrogen-containing bisphosphonates (N-BPs) preventing post-transitional protein prenylation by inhibiting a key enzyme in the mevalonate pathway in these cells (see Figure 4 for exact mechanism). Studies of the potency of N-BPs showed that the N-BP with the greatest antiresorptive potency (heterocyclic-containing N-BP's) reduced J774 cell viability the most [50,73]. This provided the first evidence that nitrogen containing bisphosphonates can successfully modify macrophages *in vitro*.

Further studies explored how differences in R2 side chain of N-BPs could affect their potency, and showed that FPP synthase was inhibited by N-BPs in a dose-dependent fashion, with the following order of potency: ZOL > RIS > IBA > ALN > PAM [53]. These findings are supported by data from J774A.1 macrophage-like cells showing the same order of potency of the drugs in relation to induction of apoptosis [49].

In addition to inhibiting FPP, N-BPs have the unique ability to evoke the production of ApppI, which inhibits the ability of mitochondrial ANT leading to excess IPP accumulation and consequently cell apoptosis. The order of potency of ApppI production in J774 macrophages is ZOL > RIS > IBA > ALN, whereas clodronate, a simple BP does not induce ApppI production [51]. Taken together, ZOL is shown to be the most potent inducer of apoptosis in macrophages *in vitro*, as expected due to its superior ability to evoke ApppI production and inhibit FPP synthase.

#### Effects on proliferation

Bisphosphonates have also been shown to affect the proliferation of macrophage precursors from bone marrow cells and bone marrow-derived macrophages (BMDMs) [69]. BMDMs obtained by flushing of long bones of 6-8 weeks old mice were treated with AHBuBP (nitrogen containing but not heterocyclic BP) or AHPrBP (PAM) for 96 hours. Compared with untreated BMDMs, AHBuBP and PAM significantly inhibited M-CSF-induced proliferation of bone marrow precursors at a concentration of 0.25 μM, without evidence of cytotoxicity. Clodronate had less clear effects as there was an overlap between cytotoxicity and inhibition of cell proliferation, in agreement with reported effects of non-N-BP's on macrophage apoptosis [69]. Phagocytic monocyte cells were shown to be more sensitive to BPs compared to other cells in the haemopoietic series like granulocytes.

#### Inhibition of pro-angiogenic factors: MMP-9

MMP-9 is a matrix metalloproteinase produced by macrophages in response to stimulation by tumour-derived factors. It is involved in angiogenesis and tumour cell invasion, key processes in the metastatic cascade. Several studies have focussed on the effects of bisphosphonates on macrophage production of MMP-9 and how this has modified tumour progression [21,71,72].

Valleala *et al *investigated whether pamidronate or clodronate altered the regulation of MMP-9 in activated human monocyte/macrophages obtained from buffy coat cells of healthy volunteers [71]. Macrophages were pre-treated for 20-24 hours with CLO (3- 1000 μM) or PAM (1- 300 μM) and then activated with LPS (lipopolysaccharide) to increase the expression of inflammatory cytokines including MMP-9 [71,74]. CLO significantly inhibited MMP-9 expression in a dose-dependent fashion at concentrations of 30-1000 μM, as did PAM at concentrations of 100-300 μM. The authors suggest that PAM had two effects on macrophages, firstly it increased MMP mRNA stability, thereby increasing MMP message levels, accounting for the increase in MMP-9 expression at lower concentrations; secondly, the higher concentrations inhibited protein prenylation enough thus decreasing MMP-9 expression. This study shows that bisphosphonates have an effect on macrophage MMP-9 expression *in vitro*.

MMP-9 inhibition by N-BPs may also have consequences for myeloid-derived suppressor cell expansion and macrophage infiltration *in vivo*. The MMP-9 - VEGF loop not only supports angiogenesis and invasion but "forces hyperactive haematopoiesis" which aids tumour progression [21,38]. Moreover, VEGF causes expansion of myeloid-derived suppressor cells (a population characterised by immature macrophages, granulocytes and dendritic cells) that are capable of suppressing T-lymphocyte proliferation and T-cell activation; thus they provide immunosuppression by the tumour [75-77]. In addition to TAMs, myeloid-derived suppressor cells have been described as one of the main antagonists of immunotherapies [76].

To investigate the effects of N-BPs on the relationship between MMP-9, TAMs and MDSCs *in vivo*, BALB-neuT mice expressing activated mouse mammary tumour virus (rat c-erbB-2/neu transgene) were treated for 5 days per week with ZOL (0.1 mg/kg) or PAM (2 mg/kg,) [21]. Treatment was started at 3 different points: 4 weeks (pre-hyperplastic), 7 weeks (hyperplastic) and 12 weeks (detectable in-situ mammary carcinomas), and continued until week 28. In mice treated from 4 or 7 weeks, PAM and ZOL delayed tumour onset, decreased tumour volume and number of transformed mammary glands. In mice receiving treatment from 12 weeks, PAM and ZOL decreased overall tumour volume. ZOL decreased macrophage infiltration into the tumour stroma associated with significantly decreased levels of serum pro-MMP-9 and VEGF in all groups. This decreased MDSC expansion in bone marrow and peripheral blood, and consequently decreased immunosuppression. In support of these results, ZOL improved immunotherapy outcome in FVB-BALB-neuT mice that had received a plasmid DNA vaccine, decreasing overall tumour volume and MDSC expansion.

Interestingly, Melani *et al *showed that ZOL appeared to act preferentially on tumour-enhanced but not normal haematopoesis. The number of colonies in bone marrow cells in BALB/c mice treated for 16 weeks with ZOL and control mice were nearly identical. However, decrease in MMP-9 and VEGF at the tumour site did not impair angiogenesis, suggesting the presence of a bypass route e.g. up-regulation of fibroblast growth factor, although this remains to be established [21]. ZOL has also been shown to be effective at suppressing macrophage MMP-9 expression in models of prostate [78], and cervical carcinoma [72].

Importantly, these studies provide evidence that targeting the production of MMP-9 within the tumour microenvironment is a successful alternative to trying to inhibit MMP-9 itself that has failed or resulted in toxicity [21,71,72,78].

#### Inhibition of pro-angiogenic factors in clinical studies

VEGF is a major pro-angiogenic cytokine produced by both tumour cells and TAMs and clinical studies have focussed on how N-BP's affect VEGF serum levels over time. Santini *et al *investigated the effects a single infusion of PAM could have on circulating VEGF levels over 7 days. 25 patients with advanced solid tumour and bone metastasis were infused with 90 mg of PAM and their VEGF levels were measured 1, 2 and 7 days after administration. The greatest significant decrease in VEGF levels occurred 2 days after administration and continuing up to 7 days post-administration; while these results are interesting, this study did not include a cancer-free control cohort [79]. The same group has also determined effects of 4 mg of ZOL in patients with advanced solid cancer and bone metastasis and measured their VEGF (and PDGF) levels 1, 2, 7 and 21 days after ZOL administration [80]. ZOL significantly decreased circulating VEGF levels from 2 days after administration and the effect was maintained until day 21. PDGF is another pro-angiogenic factor expressed by TAMs, although it is less potent than VEGF. PDGF levels fell significantly 1 and 2 days after ZOL administration but that it returned to basal level by day 7 [79,80]. Similar findings are reported using the same drug regime in advanced breast cancer patients; a significant reduction in circulating VEGF level at all time-points (1, 2, 7 and 21 days after ZOL administration); moreover the most significant decrease occurred 21 days after ZOL administration with over half the patients showing a minimum of 25% reduction in circulating VEGF [81].

These studies indicate that despite 4 mg of ZOL being cleared from the circulation within hours of administration, the effects on VEGF serum levels are still significant several weeks later. ZOL is slowly released from bone into the circulation over the course of around 7 days, which could account for the continued reduction of VEGF at this point. However, how ZOL is still modifying VEGF levels at day 21 and which cell types that are involved remains to be established [80-82]. Importantly, there was no correlation between ZOL induced VEGF reduction and increased survival compared to patients who did not experience a VEGF reduction in this small study, demonstrating ZOL is not the complete anti-cancer agent [81].

To determine whether more frequent dosing with ZOL would cause a greater decrease in circulating VEGF, effects of repeated low-dose therapy (metronomic drug regime) on VEGF levels were investigated [82,83]. Patients with advanced solid cancer and bone metastases received 1 mg of ZOL once a week for four weeks followed 4 mg every 28 weeks (three times). VEGF levels were measured at day 0 and again at 7, 14, 21, 28, 56 and 84 days. The results showed that these low doses did indeed significantly decrease serum VEGF levels at all time points.

The reports discussed above have shown that N-BP's significantly affect three pro-angiogenic factors like MMP-9, VEGF and PDGF. However the cell type responsible is unknown, and while these factors can all be expressed by TAMs, they can also be expressed by tumour cells and other stromal cells. However, these studies important as they clearly demonstrate that treatment with ZOL alone may affect tumour growth outside the skeleton.

#### Reversing M2 polarisation

Using a murine model of mammary carcinoma, Coscia *et al *investigated the cellular effects of decreased VEGF levels caused by clinically achievable doses of ZOL [83]. As discussed above, VEGF is one of the foremost factors instigating the polarisation of macrophages from the M1 to the M2 phenotype. A few papers have focussed on the effects of restoring M1 phenotype and the potential anti-tumour results gained form this. Starting at 7 weeks of age, BALB-neuT mice were treated with 100 μM/kg of ZOL once a week for four weeks followed by 3 weeks rest; with the average mouse receiving 16 injections. ZOL treated mice showed significant increase in tumour-free survival and overall survival, as well as significant reduction in tumour growth rate and tumour multiplicity, in comparison to control [77]. Histological analysis showed significant decrease in the size of lung metastases in ZOL treated mice compared to control, and immunohistological staining showed that ZOL treated mice had impaired TAM recruitment and infiltration into tumour stroma as well as clearly reduced neo-vascularisation. Tumours from control mice had significantly more intracytoplasmic VEGF staining (in TAMs and tumour cells) compared to ZOL treated mice this correlated to both a decrease in TAM density and in serum VEGF levels. This is the first study showing that ZOL reverses the polarity of peritoneal and tumour-associated macrophages from M2 to M1. Macrophages isolated from ZOL treated mice expressed iNOS, a protein considered the hallmark of M1 polarisation, whereas control mice did not. This is a very important finding, as M1 macrophages possess tumoricidal activity, supporting that TAMS are a potential immune target of ZOL therapy [8,83,84]. Moreover these data show that BPs have a clear effect on tumour macrophages *in vivo *following clinically relevant dosing.

### Effect of bisphosphonate-induced macrophage depletion on tumour growth *in vivo*

This review focuses mainly on the roles of macrophages and the effect of bisphosphonates in the tumour microenvironment in breast cancer; however effects on TAMs have also been reported in other tumours. Bisphosphonate-induced macrophage depletion has been the focus of a number of studies using animal models of metastatic lung cancer, metastatic liver cancer, melanoma and others (see Table 3 and 4). In these studies, macrophages were mainly targeted using clodrolip; a formulation of liposome encapsulated clodronate (Cl_2_MDP-LIP) [85-88].

**Table 3:** Summary of *in vitro *studies reporting effects of bisphosphonate on macrophages

Cell Type	Bisphosphonate	Main Findings	Reference
J774 cells RAW 264 cells	PAM, ALN, IBA, 100 μM 24 hours	Inhibited macrophage proliferation. Reduced cell viability. Increased cell death.	Rogers *et al *[68]
J774 cells	ALN, 25 μM or 100 μM IBA 5 μM, 7.5 μM or 10 μM 24 hours	Dose dependent increase in accumulation of unprenylated Rap1A.	Firth *et al *[97]
RAW 264 cells	ALN 10 μM; 5, 7, 9 or 16 hours ALN 100 μM; 1, 3, 5, 7, 9, 16 hours.	Dose and time dependent increase in accumulation of unprenylated Rap1A. Detectable after 16 hours incubation with 10 μM or 5 hours incubation with 100 μM	Monkkonen *et al *[98]
J774 A.1 cells	PAM, ZOL, ALN, RIS 1-100 μM for 72 hours.	All BPs induced significant apoptosis ZOL > RIS > ALN > PAM.	Moreau *et al *[49]
Macrophage precursor from bone marrow cells and bone marrow derived macrophages	PAM 2.5 × 10^-7^M (=0.25 μM), 96 hours	Significant inhibition M-CSF induced proliferation of bone marrow precursors	Cecchini *et al *[69]
Activated human monocyte/macrophage	PAM 100-300 μM 24 hour pre-treatment Then activated with LPS.	Dose-dependent inhibition of MMP-9 expression Lower doses PAM increases expression.	Valleala *et al *[71]
Human macrophage-like cell line U937	Clodrolip 20-200 μM 2 days	Decreased cell survival	Hiraoka *et al *[85]
Murine Macrophages	Clodrolip 9 μg per well 29 hours	Decreased cell viability	Gazzangia *et al *[86]
Murine peritoneal macrophages	Clodrolip 1 mg/ml 6 hours.	Dose-dependent increase in apoptosis.	Zeisberger *et al *[87]
Bone marrow cells from naive mice cultured with M-CSF or tumour supernatant.	Zoledronic acid 0.03, 0.15, 0.3 μM 6 days	Dose-dependent inhibition in differentiation of myeloid cells to macrophages. Decreased in M2 phenotype compared to control.	Veltman *et al *[89]

**Table 4:** Summary of *in vivo *studies investigating bisphosphonate effects on macrophages.

Model	Bisphosphonate	Main Findings	Reference
BALB-neut mice with mammary tumour virus (rat c-erb-2-neu/transgene)	ZOL 0.1 mg/kg or PAM 2 mg/kg 5 days a week.	Zol decreased macrophage infiltration into tumour stroma associated with decreased levels of pro-MMP-9 and VEGF	Melani C *et al *[21]

Mammary carcinoma cells implanted in BALB-neutT mice	ZOL 100 μM/kg Once a week for 4 weeks, followed by 3 weeks rest, cycle continued.	Impaired TAM recruitment and infiltration into tumour and reduced neo-vascularisation reversal of TAM polarity from pro-tumoural M2 to tumoricidal M1	Coscia *et al *[83]

HARA-B lung cancer cells implanted in BALB/c nude mice	Clodrolip 200 μL or 400 μL Every 3 days for 6 weeks (s.c.).	Reduced TAM infiltration correlated to reduced metastatic spread	Hiraoka *et al *[85]

Human melanoma cell line IIB-MEL-J with or without MCP-1 expression vector. Athymic male NIC-(S)-Nu mice	Clodrolip 50 μl or 200 μl (6 mg clodronate per 1 ml ) From day before cell injection and every 5 to 7 days thereafter.	Reduced TAMs infiltration correlated to decreased tumour volume and angiogenesis and increased survival	Gazzangia *et al *[86]

F9 teratocarcinoma cells implanted in SV129 female mice	Clodrolip, 1 mg/20 g every 4 days.	Reduced TAM infiltration. 82% reduction in tumour volume	Zeisberger *et al *[87]

A673 rhabdomyosarcoma cells into CD-1 nude mice	Clodrolip, 1 mg/20 g every 4 days.	93% reduction in TAM 89% reduction in blood vessel density	Zeisberger *et al *[87]

Metastatic liver cancer Mouse model LM3R or SMMC7721 human hepatocellular cell lines in BALB/c nu/nu mice	Clodrolip 100 μg/kg 3 times a week or, ZOL 100 μg/kg 3 times a week and or 30 mg/kg sorafenib.	Reduced TAM infiltration with combination therapy. Correlated with decreased tumour growth, angiogenesis and lung metastasis. ZOL had greater effect than clodrolip	Zhang et al [88]

Cervical carcinoma K14-HPV16 transgenic mice	ZOL 100 μg Every day for 3 months.	Decreased MMP-9 expression by TAMs	Giraudo *et al *[72]

Peritoneal macrophage obtained from CBA-J mice injected with AC29 mesothelioma cells	Clodronate 200 μl twice over 10 days.	Depleted peritoneal macrophages. Decreased tumour growth	Veltman *et al *[89]

CBA-J mice injected with AC29 mesothelioma cells	ZOL 100 μg/kg s.c. Every day for 25 days.	Increased myeloid precursors. Decreased TAMs No significant increase in survival or decrease in tumour burden	Veltman *et al *[89]

#### Macrophage depletion using Clodrolip alone

Hiraoka *et al *investigated the effects of clodrolip on macrophage infiltration in BALB/c nude mice injected with HARA-B lung cancer cell line, focusing on the effect on bone and muscles metastasis [85]. Mice were treated with either clodrolip (200 μL or 400 μL once every three days for 6 weeks) 10 mg/kg reveromycin A daily for 6 weeks, or a PBS control. Clodrolip treated mice had significantly reduced bone metastasis. Immunohistochemical analysis of the tumours showed decreased macrophage infiltration in clodrolip treated mice in comparison to mice receiving PBS or reveromycin A. The HARA-B lung cancer cells were not affected by clodrolip *in vitro*, supporting that the effect of clodrolip on metastatic spread was due to its actions on macrophages rather than on the lung cancer cells [85].

#### Clodrolip in combination with anti-cancer agents

The effects of clodrolip combined with MCP-1 inhibition have been investigated on tumour growth and angiogenesis in a melanoma model. One day after the first clodrolip treatment (50 μl or 200 μl), athymic male NIG(S)-Nu mice were injected with human melanoma cell line IIB-MEL-J, IIB-MEL-J-MCP-1 (IIB-MEL-J cells containing a MCP-1 expression vector) or a vehicle control. Treatment continued every 5-7 days, mice were killed on day 4 or 11. Compared to control, clodrolip caused a decrease in tumour volume greater than 70% in IIB-MEL-J-MCP mice as well as increasing their survival, and this was associated with reduced tumour angiogenesis. To identify which cell types that were targeted, melanoma and endothelial cells (intrinsic for angiogenesis) were treated *in vitro *with clodrolip, with no significant effect being observed [86]. This supports the findings of Hiraoka *et al *and confirms a direct relationship between depletion of TAMs and reduction in angiogenesis, tumour growth and increased survival [85,86].

TAM depletion has also been combined with an antiangiogenic therapy using VEGF neutralising antibody. Female Sv129 mice injected with F9 teratocarcinoma cells and CD-1 nude mice injected with A673 rhabdomyoscarcoma cells were treated with either 1 mg20 g^-1 ^clodrolip (initial dose 2 mg20 g^-1^), clodronate dissolved in phosphate buffer (67 mM) or anti VEGF Abs 0.5 mg20 g^-1 ^i.v. or clodrolip and anti VEGF Abs in combination. F9 mice treated with clodrolip had an 82% reduction in tumour volume, while those treated in combination had 92% reduction. Similar results were obtained in A673 mice. Immunohistochemical staining of tumours from A673 animals showed a significant reduction in blood vessel density (CD31+) (89% with clodrolip alone and 85% in combination therapy), this reduction was significant up to 9 days after the end of therapy. Clodrolip-treated animals also exhibited a 93% depletion of TAMs which was not enhanced by combination treatment. As shown for other tumour cell types clodrolip had no effect on F9 or A673 *in vitro*, supporting that the effects on tumour volume were due to TAM depletion [87].

The multi-targeted kinase inhibitor sorafenib has been combined with clodrolip or ZOL in models of metastatic liver cancer. The human hepatocellular cancer cell lines LM3R and SMMC7721 were orthotopically implanted into the liver of BALB/c nu/nu mice, and animals were treated with sorafenib (30 mg/kg daily), clodrolip (100 μg/kg three times per week), ZOL (100 μg/kg three times per week), or sorafenib combined with clodrolip or ZOL. Mice treated with sorafenib alone showed increased tumour macrophage infiltration and peripheral blood monocytes compared to control, however, mice receiving ZOL or clodrolip alone showed no significantly suppressed infiltration and decreased peripheral blood monocytes. Mice receiving ZOL or clodrolip alone showed no significant difference in either compared to control. Overall, combination treatment caused significantly decreased tumour growth, decreased angiogenesis, and decreased lung metastasis [88].

#### Differential effects of ZOL and clodrolip in vivo

The effects of ZOL differentiation of macrophages from myeloid cells to TAMs has been studies in CBA-j mice injected with AC29 mesothelioma cells [89]. Mice receiving 2 doses of intraperitoneal clodrolip (day 5 and 10) had significantly decreased numbers of macrophages in the peritoneal cavity. Tumour growth was reduced in all treated animals on day 12, with 60% of the mice failing to develop tumours, demonstrating that macrophages play a significant role in tumour development in this model. In the same study, mice were treated daily with 100 μg/kg ZOL for 25 days following implantation of AC29 cells. ZOL induced reversal of TAM polarisation from the M2 e to the M1 phenotype, and higher numbers of myeloid precursors and lower numbers of TAMs were detected in ZOL treated mice compared to control. However, tumour burden and survival were unaffected by ZOL. The authors hypothesised that ZOL treatment may result in up-regulation of a sub-population of immature myeloid cells with immunosuppressive properties [89]. Despite using a very intensive ZOL schedule (equivalent to the 4 mg clinical dose given daily for several weeks), this study failed to demonstrate a significant anti-tumour effect.

#### Optimising ZOL delivery using nanothechnology

Recent studies have aimed at optimising the delivery of ZOL using nanotechnology, in an attempt to overcome the limitations of its pharmacokinetic properties and thus extend the exposure time of extra-skeletal tumours to these agents [90-92]. Two delivery systems have been developed where ZOL is either encapsulated in stealth liposomes or in PEGlyated nanoparticles. Administration of stealth liposomes (LIPO-ZOL) was well tolerated and found to significantly reduce tumour growth and increase survival in mouse models of human prostate cancer and multiple myeloma [93]. The second approach involved encapsulation of ZOL in PEGylated particles of calcium phosphate (PLCaPZ NP), and two forms, pre-PLCaZ NP and post-PLCaZ NP, have been tested both *in vitro *and *in vivo *[92]. First the effects of PLCaPZ NPs on growth *in vitro *was determined in a number of cancer cell lines including prostate, breast, lung, pancreas and multiple myeloma. Pre-PLCaPZ NPs had highest anti-proliferative effect, and was subsequently tested in an *in vivo *model of prostate cancer. CD-1 nude mice were injected with human prostate cancer cells (PC-3) and divided into groups receiving either control, blank NPs, free ZOL or pre-PLCaPZ NPs (0.25 mg/ml) three times per week for three weeks. Animals treated with PLCaPZ NP showed 45% tumour weight inhibition and a tumour growth delay of 10 days, and this was significantly greater than in mice treated with free ZOL [92]. In a recent study comparing LIPO-ZOL to PLCaPZ NPs and free ZOL, CD-1 male nude mice were injected with human prostate cancer cells and placed in one of six treatment groups: untreated, empty NPs, empty liposomes, free ZOL, LIPO-ZOL (108 μg/ml) and PLCaNPs (66 μg/ml) and treated three times a week for three consecutive weeks [91]. PLCaNZ NPs delayed tumour grown by 12 days, significantly longer than LIPO-ZOL (7 days) and free ZOL (3 days). Moreover, PLCaPZ NP caused 65% necrotic index and also induced strong anti-angiogenic effects, both significantly greater than compared to LIPO-ZOL [91]. Most interesting in regard to this review was the immunohistochemical staining showing tumours of LIPO-ZOL and PLCaPZ NP treated animals had 28% and 18% of TAMs, respectively. This was significantly less than the numbers found in controls or animals receiving free ZOL. Encouragingly, ZOL only affected TAMs and the presence of macrophages in normal tissues was not altered [91].The combination of anti-tumour and anti tumour-associated macrophage effects of PLCaNZ NPs may be due to this delivery method resulting in a longer elevation of plasma ZOL. Further studies with other tumour types *in vivo*, especially breast cancer models, would be of interest.

## Conclusions

Bisphosphonates are routinely used in the treatment of cancer-induced bone disease, and there is a growing body of evidence both *in vitro *and *in vivo *supporting their potential anti-tumour activity, mainly from studies focussing on effects of tumour cells. However, as summarised in this review, bisphosphonates may also affect tumour growth by modifying cells of the tumour microenvironment. Bisphosphonates induce macrophage apoptosis *in vitro *and to inhibit the release of pro-angiogenic factors, and there is evidence showing that bisphosphonates can affect tumour macrophages *in vivo *by reversing their polarity to tumoricidal phenotype. Whereas data from model systems suggest that effects on macrophages in tumour microenvironment could contribute to the bisphosphonate anti-tumour effects, whether macrophages could be a target of these agents following clinical dosing remains to be determines. To firmly establish the potential for targeting of tumour macrophages with bisphosphonates requires *in vivo *studies using clinically relevant dosing regimens as well as analysis of TAMs in tumour material from neoadjuvant studies of patients receiving bisphosphonate treatment.

## List of Abbreviations Used

ADP: Adenosine diphosphate; ALN: Alendronate; ANG: Angiopoietin; ApppI: New endogenous ATP analog; BP: bisphosphonate; CCL: CC chemokines; CIBD: Cancer Induced Bone Disease; CLO: Clodronate; COX2: Cyclooxygenase-2; CXCL: Chemokine Interleukin; CSF: Colony Stimulating Factor; DCIS: Ductal Carcinoma In Situ; DOX: doxorubicin; ECM: Extracellular matrix; EGF: Epidermal Growth Factor; EL: Endothelin; ETI: Etidronate; FGF2: Fibroblast Growth Factor 2; FFP: Farnesyldiphosphonate; HGF: Hepatocyte growth factor; HIF: Hypoxia-Inducible Transcription Factor; IBA: Ibandronate; IL: interleukin; IL: 1ra- IL-1 receptor antagonist; INFγ: Interferon-gamma; iNOS: Inducible NO synthase; LCIS: Lobular Carcinoma in Situ; LPS: Lipopolysaccharide; MDSC: Myeloid Derived Suppressor cell; MCP-1: Monocyte Chemotactic Protein - 1; MMPs: Matrix Metalloproteinases; NK: Natural killer cells; N-BP: Nitrogen containing bisphosphonate; NO: Nitric Oxide; PAM: Pamidronate; PDGF: Platelet-Derived Growth Factor; PGE_2_: Prostaglandin E_2_; PyMT: Polyoma middle T oncoprotein; RANK: Receptor Activator of Nuclear Factor κ; RANKL: Receptor Activator of Nuclear Factor κ Ligand; RIS: Risedronate; SCID: Severe Combined Immunodeficiency; SRE: Skeletal Related Event; TAM: Tumour Associated Macrophages; TEM: Tumour Educated Macrophage; TGF-β: Transforming Growth Factor - Beta; TLR: Toll-like receptor; TNF-α: Tissue Necrosis Factor Alpha; VEGF: Vascular endothelial growth factor; VEGFR: Vascular endothelial growth factor receptor

## Competing interests

The authors declare they have no competing interest.

## Authors' contributions

TLR was the main author of the body text, carried out literature searches and designed the figures. IH conceived the idea for the manuscript, contributed to the body text and adjusted the figures. Both authors contributed equally to the work and approved the manuscript prior to submission.

## Authors' information

IH is reader in Bone Oncology at the University of Sheffield Medical School, specialising in studies of anti-cancer agents in advanced breast and prostate cancer. TLR has carried out studies of the effects of bisphosphonates on macrophages supervised by IH.
